# Editorial: Toward and beyond human-level AI, volume II

**DOI:** 10.3389/fnbot.2022.1120167

**Published:** 2023-01-06

**Authors:** Witali Dunin-Barkowski, Alexander Gorban

**Affiliations:** ^1^Department of Neuroinformatics, Center for Optical Neural Technologies, Scientific Research Institute for System Analysis, Russian Academy of Sciences, Moscow, Russia; ^2^Department of Mathematics, University of Leicester, Leicester, United Kingdom; ^3^Scientific and Educational Mathematical Center “Mathematics of Future Technology,” Lobachevsky State University of Nizhny Novgorod, Nizhny Novgorod, Russia

**Keywords:** artificial intelligence, strong AI, learning to learn, real time learning, sample-efficient learning

AI systems have surpassed human-level in a lot of computational competencies, and the number of these competencies is growing almost daily. Still, modern AI systems are specialized gadgets which are aimed at solving specific particular problems or limited sets of problems. The universal human type intellectuality for these systems still remains unattained. In the opinion of many professionals, obtaining practical solution for that general problem seems to be very hard (Zhang et al., 2022) and it is not clear when it can be achieved (Stein-Perlman et al., 2022). Also, this task seems to be tough for general public (Schmidt, 2022).

In the first paper of this topic (Rivera, Popek et al.), authors explore a novel framework called PICO to detect presence of novel behaviors and construct a library of behavior primitives from unlabeled demonstrations. A gap in trajectory is defined as a region where actions cannot be predicted with high enough probability in a current behavior model. When such a gap occurs a new behavior primitive is added. The approach is evaluated using a reach-grab-lift task using a robotic arm, showing better label and reconstruction accuracies when compared to similar approaches.

The paper Rivera, Staley et al. is devoted to multi-agent reinforcement learning in complex environments such as dense urban defense-related scenarios. The authors introduce the AI Arena framework. There, different agents control tanks and fight with each other in a 5 vs. 5 tank combat game. It is shown that agents of the same team converge to a cooperative set of behaviors. Thus emergence of cooperative behavior has been demonstrated in the work.

In the third paper (Limbacher and Legenstein), authors demonstrated the emergence of clustering of temporally correlated inputs on dendritic branches in a setting with a generic stochastic rewiring principle with a simple synaptic plasticity rule. The mechanism is demonstrated in a computational model and is backed up with a heavy theoretical analysis. The hypothesis is proposed that such clustering might serve to protect memories from catastrophic forgetting on a medium time scale.

Finally, Krauss and Maier gives a brief review on theories of consciousness in neuroscience and AI. The general philosophical perspective on the problem is given, the main directions in philosophy of consciousness are described. Some noteworthy experiments in neurophysiology of consciousness are reviewed. Schmidhuber, 1991 as well as other popular theories of consciousness are discussed. Overall, many interesting experiments, theories and ideas are described in the paper.

Still it is not clear what can be done to achieve the goal of AGI. One of the most significant problems in moving toward general intelligence is the speed of learning. In this respect, a topic of special importance is one or few-shot learning. This kind of learning really works in practice in technical and biological systems, despite the apparent contradiction to what classical statistical theory states. It turns out that it is possible to find a general approach to the modern mathematical formulation of the problem (Tyukin et al., 2021a). In some cases it is possible to show the relationship between intrinsic dimensions of the transformed data and the probabilities to learn successfully from few demonstrations (Gorban et al., 2021b; Tyukin et al., 2022).

The problem of learning from a small number of examples is strongly related to the recently discovered phenomenon of dimensionality blessing (Gorban et al., 2016) as opposed to the “curse of dimensionality” (Sutton et al., 2022). The connection between these two problems has a fundamental mathematical nature (Gorban and Tyukin, 2017). In particular it is connected to the Hilbert's sixth problem (Gorban and Tyukin, 2018) and the fact of surprising effectiveness of small neural ensembles in high-dimensional brain (Gorban et al., 2019). These properties can be effectively applied to concrete features of the live brain hippocampus (Tyukin et al., 2019).

The further analysis reveals the existence of a fundamental tradeoff between complexity and simplicity in high-dimensional spaces (Gorban et al., 2020) and effectively uses the geometry of few-shot learning (Tyukin et al., 2021c). Among the important tasks for creating practical AI, it is important to note the development of new methods (Akinduko et al., 2016; Mirkes et al., 2022; Zhou et al., 2022) and tools (Rybnikova et al., 2020, 2021; Bac et al., 2021) for creation of AI systems.

In search of AGI one of the most important clusters of problems is connected with medical applications. Even in such seemingly routine problems as child health monitoring (Roland et al., 2021) there can be inherent obstacles. In the more complicated cases of psychological profiling within the context of drug abuse avoidance machine learning yields impressive results (Fehrman et al., 2015). The general consideration of adaptability of individuals and technical complexes also yields useful hints for solving the problem of AGI (Gorban et al., 2021a). Finally, the safety of AI systems should be prioritized without any doubts (Tyukin et al., 2021b).

The grand modern trend in developing AI systems is coming back to attempts to understand mechanisms of real brain functioning as exposed in recent Nature editorial (Mehonic and Kenyon, 2022) and in appeal of world prominent researchers in AI and computational neuroscience (Zador et al., 2022). There it has been argued that much more studies of natural neural intelligence need to be done for obtaining real general intelligence in technological systems. In this respect, the role of glia in information processing in the brain has been revealed in thorough studies (Gordleeva et al., 2019). Also more attention is needed toward probably unduly overlooked ideas about the operation principles and functions of the cerebellum (Dunin-Barkowski and Wunsch, 2000; Shakirov, 2022). Besides motion control the cerebellum deals with human emotions (Adamaszek et al., 2022) and with organism's rewards (Kostadinov and Häusser, 2022) etc. It deals with many cognitive problems especially of those connected with vision (Vaina et al., 2001) including creative tasks (Saggar et al., 2015). Unfortunately, even the crude details of cerebellar circuit operation present the subject of disagreement between theoreticians (Willshaw et al., 2015) and experimentalists (Streng et al., 2018).

## Author contributions

All authors listed have made a substantial, direct, and intellectual contribution to the work and approved it for publication.
